# Supplementary material for: Stimulation Parameters Recruit Distinct Cortico-Cortical Pathways: Insights from Microstate Analysis on TMS-Evoked Potentials
Source: Brain Topogr. 2025 Mar 28;38(3):39. doi: 10.1007/s10548-025-01113-2 (PMC11953218; doi:10.1007/s10548-025-01113-2)
Supplement: Supplementary file 1 — Supplementary Material 1 [file 10548_2025_1113_MOESM1_ESM.docx]

**Stimulation parameters recruit distinct cortico-cortical pathways: insights from microstate analysis on TMS-evoked potentials**

Delia Lucarelli, Giacomo Guidali, Dominika Sulcova, Agnese Zazio, Natale Salvatore Bonfiglio,

Antonietta Stango, Guido Barchiesi & Marta Bortoletto

**- SUPPLEMENTARY MATERIALS -**

**SUPPLEMENTAL FIGURES**

**
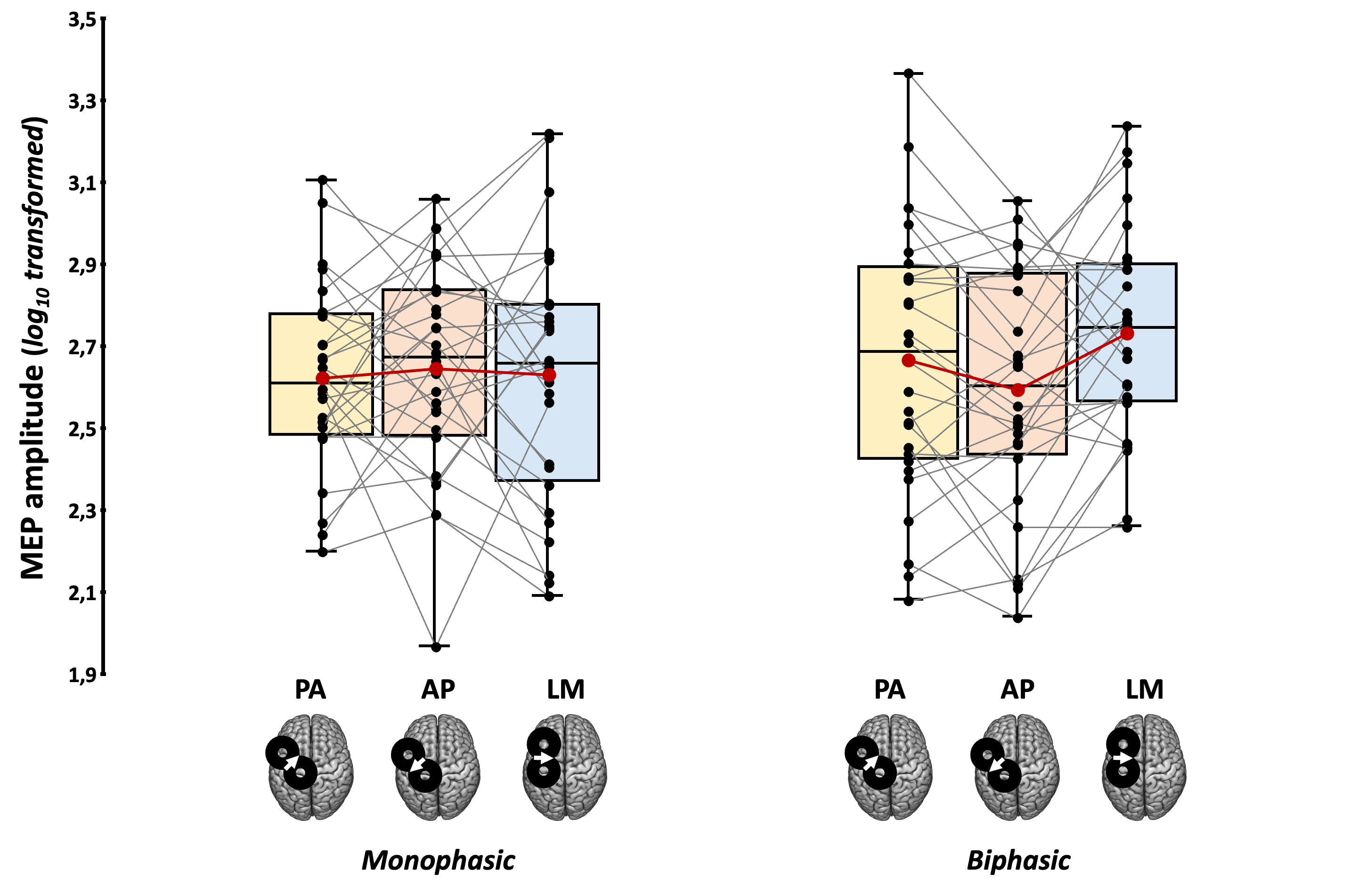
**

**Supplemental Figure 1.** Log_10_-transformed MEP amplitude recorded in the six experimental conditions in the original study of Guidali et al. (2023). Data were analyzed with a within-subject repeated-measures analysis of variance (rm-ANOVA) with factors ‘Pulse waveform’ (monophasic, biphasic) and ‘Current direction’ (PA, AP, LM). No main effect was detected for factors ‘Pulse waveform’ (F_1,27_ = 1.39; *p* = .249; ƞ_p_^2^ = .049) and ‘Current direction’ (F_2,54_ = 1.46; *p* = .242; ƞ_p_^2^ = .051), and their interaction (F_2,54_ = 2.76; *p* = .072; ƞ_p_^2^ = .093). In the box-and-whiskers plots, red dots and lines represent the means of the distributions. The centre line depicts their median values. Black dots and grey lines show individual scores. The box contains the 25th to 75th percentiles of the dataset. Whiskers extend to the largest observation, which falls within the 1.5 times interquartile range from the first/third quartile. For further information see: Guidali et al., 2023.


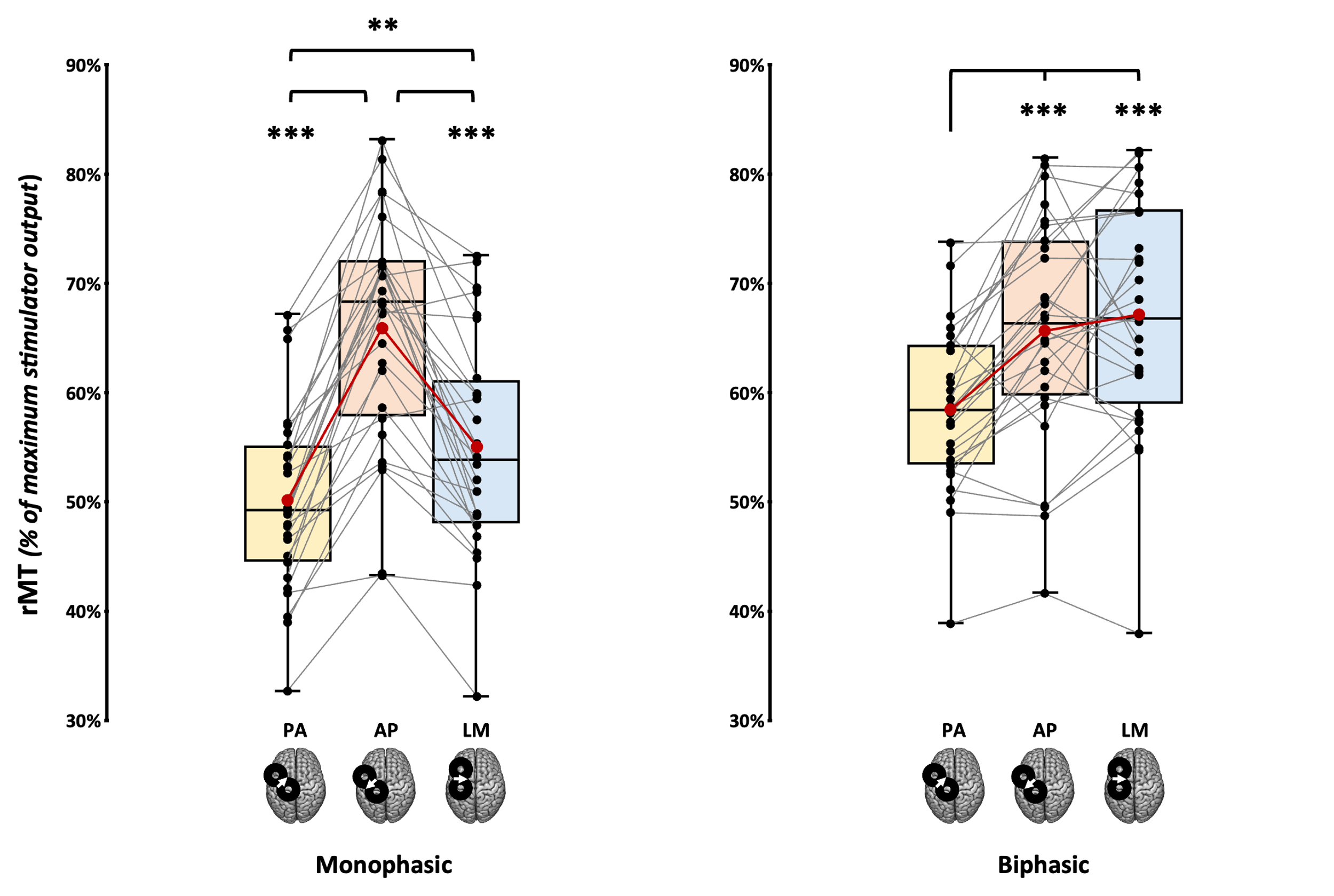


**Supplemental Figure 2.** rMT values recorded in the six experimental conditions in the original study of Guidali et al. (2023). Given the different maximum discharge energy of the two stimulators used (i.e., approximately 725 for Magstim 200 *vs.* 250 for Magstim Rapid 2 - Deng et al., *Clinical Neurophysiology*, 2014), we analysed rMT separately for monophasic and biphasic waveforms. Rm-ANOVAs showed significant effects of ‘Current direction’ for both monophasic (F_2,54_ = 60.46; *p* < .001; ƞ_p_^2^ = .69) and biphasic conditions (F_2,54_ = 25.44; *p* < .001; ƞ_p_^2^ = .49). In the box-and-whiskers plots, red dots and lines represent the means of the distributions. The centre line depicts their median values. Black dots and grey lines show individual scores. The box contains the 25th to 75th percentiles of the dataset. Whiskers extend to the largest observation, which falls within the 1.5 times interquartile range from the first/third quartile; significant p-values of corrected Tukey’s post hoc comparisons are reported (** = p <.01; *** = p <.001). For further information see: Guidali et al., 2023.

**
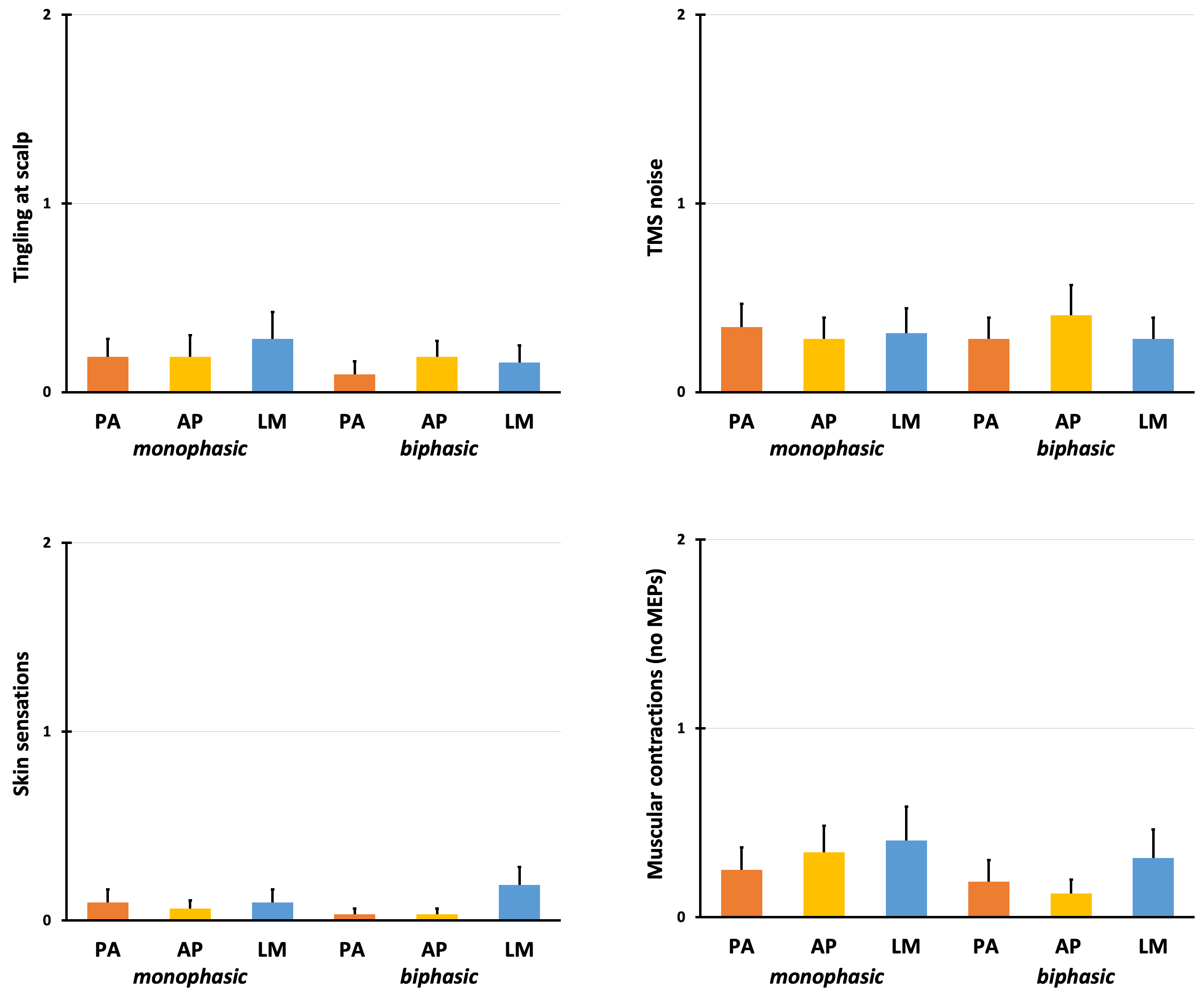
**

**Supplemental Figure 3**. Responses at the questionnaire administered in the original study of Guidali et al., (2023) investigating the presence and the discomfort degree of TMS-related peripheral sensations (i.e., tingling at scalp, TMS noise, skin sensations, and muscular contractions - excluded ‘targeted’ MEPs). The questionnaire, adapted from Giustiniani et al., (2022, Clinical Neurophysiology) was administered after each stimulation conditions. In detail, for each of these sensations, we asked participants: ‘*Did you experience any of the following sensations? Please answer by inserting the number that corresponds to the degree of the experienced discomfort, with 0 (‘None’), 1 (‘Mild’), 2 (‘Moderate’), 3 (‘Considerable’), 4 (‘Strong’)*.’ Responses were analyzed through a series of Friedman’s non-parametric rmANOVA (one for each sensation) with factor ‘Condition’ (Monophasic PA, Monophasic AP, Monophasic LM, Biphasic PA, Biphasic AP, Biphasic LM). None of these rmANOVAs showed statistically significant differences across stimulation conditions (tingling at scalp: 𝒳^2^_5_= 3.58; *p* = .611; TMS noise: 𝒳^2^_5_ = 4.4; *p* = .493; skin sensations: 𝒳^2^_5_ = 4.46; *p* = .486; muscular contractions: 𝒳^2^_5_ = 5.23; *p* = .389), suggesting that TMS-related peripheral sensations perceived by participants did not significantly vary across stimulation blocks. Error bars: SE.


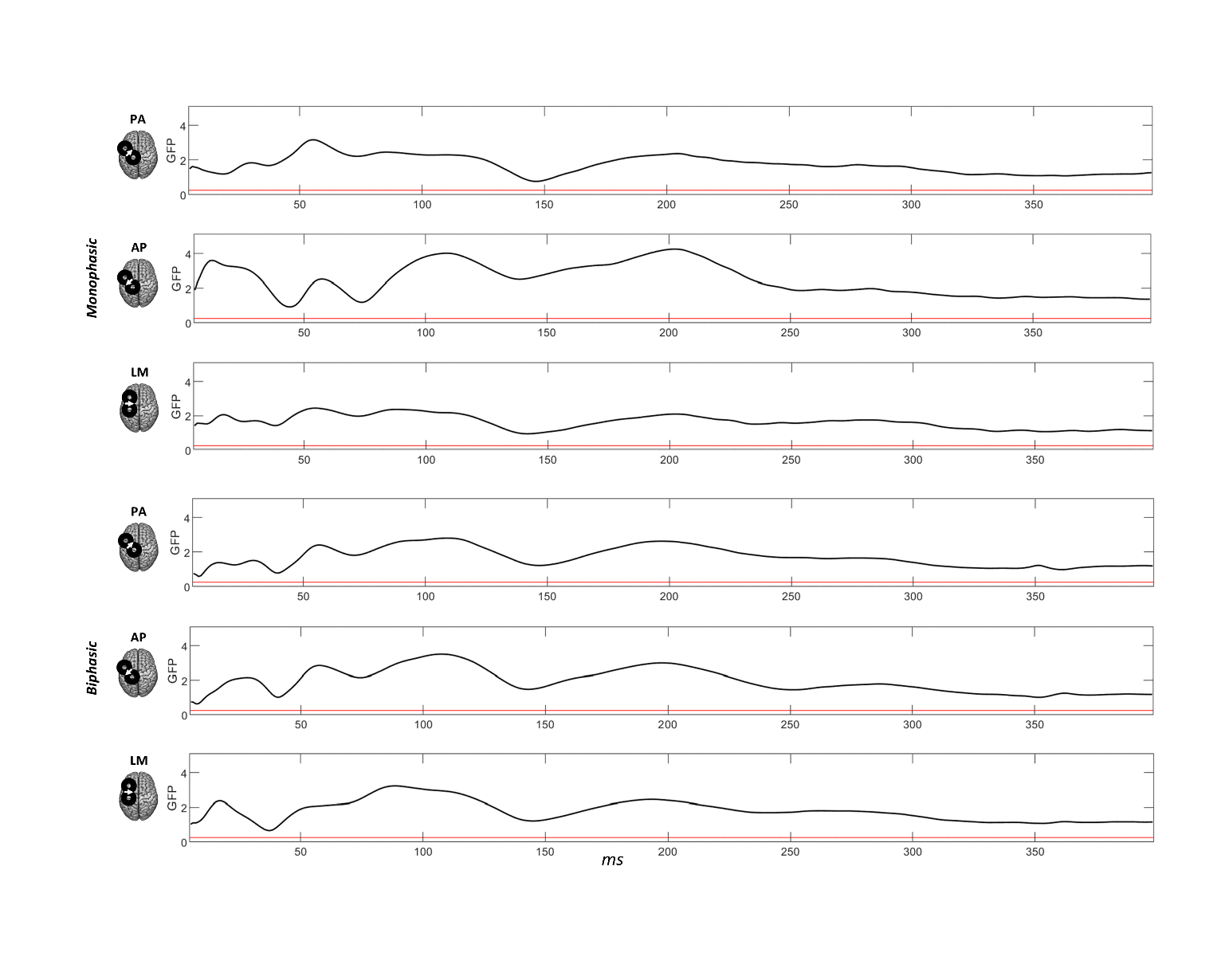


**Supplemental Figure 3.** TCT results. The black line represents the global field power of the grand average signal between 5 and 400 ms post-stimulus for each condition. The red line represents the p-value (=.05).

**SUPPLEMENTAL TABLES**

|  |  | **Monophasic PA** | **Monophasic AP** | **Monophasic LM** | **Biphasic PA** | **Biphasic AP** | **Biphasic LM** |
| --- | --- | --- | --- | --- | --- | --- | --- |
| **ICA components** | **M** | 1,96 | 2,00 | 2,05 | 1,96 | 1,96 | 1,93 |
|  | **SD** | 0,69 | 0,92 | 1,02 | 0,74 | 0,88 | 0,77 |
| **SSP-SIR components** | **M** | 1,06 | 1,14 | 1,57 | 0,56 | 0,72 | 1,13 |
|  | **SD** | 0,58 | 0,56 | 0,88 | 0,68 | 0,69 | 1,01 |
| **Artefactual epochs removed** | **M** | 1,71 | 1,69 | 1,25 | 1,47 | 1,25 | 1,4 |
|  | **SD** | 1,55 | 1,87 | 1,08 | 1,84 | 1,11 | 1,42 |

**Supplemental Table 1.** Mean (M) and standard deviation (SD) for ICA, SSP-SIR components, and artefactual epochs removed in each experimental condition.

| **TEP component** | **Outliers** | **F** |
| --- | --- | --- |
| **N15** | 3 | 15 |
| **P30** | 3 | 16 |
| **N45** | 3 | 16 |
| **P60** | 5 | 15 |
| **N100** | 3 | 16 |
| **P180** | 3 | 16 |

**Supplemental Table 2.** All the outliers identified for each TEP component. F: number of females.

| ***TEP component*** | ***Factor*** | ***F*** | ***p*** | ***ƞ_p_^2^*** |
| --- | --- | --- | --- | --- |
| **N15** | **Current direction** | **24.87** | **<.001** | **0.47** |
|  | Pulse waveform | 0.23 | .634 | 0.01 |
|  | **Current direction X Pulse waveform** | **26.53** | **<.001** | **0.49** |
| **P30** | **Current direction** | **6.42** | **.003** | **0.19** |
|  | **Pulse waveform** | **6.12** | **.02** | **0.18** |
|  | **Current direction X Pulse waveform** | **11.37** | **<.001** | **0.29** |
| **N45** | **Current direction** | **19.67** | **<.001** | **0.41** |
|  | Pulse waveform | 1.39 | .249 | 0.05 |
|  | **Current direction X Pulse waveform** | **35.98** | **<.001** | **0.56** |
| **P60** | **Current direction** | **7.14** | **.001** | **0.22** |
|  | Pulse waveform | 0.28 | .599 | 0.01 |
|  | **Current direction X Pulse waveform** | **5.29** | **0.02** | **0.17** |
| **N100** | **Current direction** | **9.28** | **<.001** | **0.25** |
|  | Pulse waveform | 3.31 | .08 | 0.11 |
|  | **Current direction X Pulse waveform** | **22.06** | **<.001** | **0.44** |
| **P180** | **Current direction** | **27.61** | **<.001** | **0.5** |
|  | Pulse waveform | 0.63 | .434 | 0.02 |
|  | **Current direction X Pulse waveform** | **19.7** | **<.001** | **0.41** |

**Supplemental Table 3.** rmANOVAs results conducted for TEP components’ amplitudes. Significant main effects and interactions are highlighted in bold.

| ***TEP component*** | ***Factor*** | | ***F*** | ***p*** | ***ƞ_p_^2^*** |
| --- | --- | --- | --- | --- | --- |
| **N15** | **Current direction** | | **10.19** | **<.001** | **.27** |
|  | **Pulse waveform** | | **5.52** | **.026** | **.17** |
|  | **Current direction X Pulse waveform** | | **10.39** | **<.001** | **.27** |
|  |  |  | |  |  |
| ***TEP component*** | ***Pulse waveform*** | ***Factor*** | | ***F*** | ***p*** |
| **P30** | Monophasic | Current direction | | 2.31 | .126 |
|  | Biphasic | Current direction | | 2.56 | .092 |
| **N45** | Monophasic | Current direction | | 0.16 | .849 |
|  | Biphasic | Current direction | | 1.12 | .338 |
| **P60** | **Monophasic** | **Current direction** | | **5.57** | **.013** |
|  | Biphasic | Current direction | | 1.65 | .21 |
| **N100** | Monophasic | Current direction | | 0.29 | .746 |
|  | Biphasic | Current direction | | 0.86 | .424 |
| **P180** | Monophasic | Current direction | | 2.15 | .131 |
|  | Biphasic | Current direction | | 0.03 | .962 |

**Supplemental Table 4.** rmANOVA (for N15) and robust rmANOVAs results conducted for TEP components’ latencies. Significant main effects and interactions are highlighted in bold.

| ***Microstate class*** | ***Pulse waveform*** | ***Factor*** | ***F*** | ***p*** |
| --- | --- | --- | --- | --- |
| **Class 1** | **Monophasic** | **Current direction** | **22.4** | **<.001** |
|  | Biphasic | Current direction | 0.65 | .53 |
| **Class 2** | Monophasic | Current direction | 1.38 | .266 |
|  | Biphasic | Current direction | 0.03 | .97 |
| **Class 3** | **Monophasic** | **Current direction** | **20.68** | **<.001** |
|  | Biphasic | Current direction | 1.55 | .229 |
| **Class 4** | Monophasic | Current direction | 0.36 | .663 |
|  | Biphasic | Current direction | 0.89 | .417 |
| **Class 5** | Monophasic | Current direction | 0.26 | .716 |
|  | Biphasic | Current direction | 1.07 | .353 |
| **Class 6** | **Monophasic** | **Current direction** | **14.08** | **<.001** |
|  | Biphasic | Current direction | 1.33 | .277 |

**Supplemental Table 5.** Robust-rmANOVAs results conducted on microstate AUC. Significant main effects and interactions are highlighted in bold.

| ***Microstate class*** | ***Factor*** | ***F*** | ***p*** | ***ƞ_p_^2^*** |
| --- | --- | --- | --- | --- |
| **Class 1** | **Current direction** | **15** | **.001** | **0.36** |
|  | **Pulse waveform** | **13.5** | **.001** | **0.34** |
|  | **Current direction X Pulse waveform** | **16.3** | **<.001** | **0.38** |
| **Class 2** | Current direction | 0.65 | .53 | 0.02 |
|  | Pulse waveform | 0.2 | .658 | 0.01 |
|  | Current direction X Pulse waveform | 0.53 | .593 | 0.02 |
| **Class 3** | **Current direction** | **19.8** | **<.001** | **0.43** |
|  | **Pulse waveform** | **7.67** | **.01** | **0.23** |
|  | **Current direction X Pulse waveform** | **18.35** | **<.001** | **0.41** |
| **Class 4** | Current direction | 0.24 | .79 | 0.01 |
|  | **Pulse waveform** | **11.74** | **.002** | **0.31** |
|  | Current direction X Pulse waveform | 0.01 | .99 | 0.00 |
| **Class 5** | Current direction | 1.04 | .361 | 0.04 |
|  | Pulse waveform | 0.06 | .816 | 0,01 |
|  | Current direction X Pulse waveform | 1.79 | .176 | 0.07 |
| **Class 6** | **Current direction** | **10.15** | **<.001** | **0.28** |
|  | Pulse waveform | 0.03 | .867 | 0.01 |
|  | **Current direction X Pulse waveform** | **10.94** | **<.001** | **0.29** |

**Supplemental Table 6.** rmANOVAs results conducted on microstate duration. Significant main effects and interactions are highlighted in bold.
